# Estimation of Respiratory Frequency in Women and Men by Kubios HRV Software Using the Polar H10 or Movesense Medical ECG Sensor during an Exercise Ramp

**DOI:** 10.3390/s22197156

**Published:** 2022-09-21

**Authors:** Bruce Rogers, Marcelle Schaffarczyk, Thomas Gronwald

**Affiliations:** 1 College of Medicine, University of Central Florida, 6850 Lake Nona Boulevard, Orlando, FL 32827-7408, USA; 2 Interdisciplinary Institute of Exercise Science and Sports Medicine, MSH Medical School Hamburg, University of Applied Sciences and Medical University, Am Kaiserkai 1, 20457 Hamburg, Germany

**Keywords:** respiratory rate, breathing frequency, heart rate variability, endurance exercise

## Abstract

Monitoring of the physiologic metric, respiratory frequency (RF), has been shown to be of value in health, disease, and exercise science. Both heart rate (HR) and variability (HRV), as represented by variation in RR interval timing, as well as analysis of ECG waveform variability, have shown potential in its measurement. Validation of RF accuracy using newer consumer hardware and software applications have been sparse. The intent of this report is to assess the precision of the RF derived using Kubios HRV Premium software version 3.5 with the Movesense Medical sensor single-channel ECG (MS ECG) and the Polar H10 (H10) HR monitor. Gas exchange data (GE), RR intervals (H10), and continuous ECG (MS ECG) were recorded from 21 participants performing an incremental cycling ramp to failure. Results showed high correlations between the reference GE and both the H10 (r = 0.85, SEE = 4.2) and MS ECG (r = 0.95, SEE = 2.6). Although median values were statistically different via Wilcoxon testing, adjusted median differences were clinically small for the H10 (RF about 1 breaths/min) and trivial for the MS ECG (RF about 0.1 breaths/min). ECG based measurement with the MS ECG showed reduced bias, limits of agreement (maximal bias, −2.0 breaths/min, maximal LoA, 6.1 to −10.0 breaths/min) compared to the H10 (maximal bias, −3.9 breaths/min, maximal LoA, 8.2 to −16.0 breaths/min). In conclusion, RF derived from the combination of the MS ECG sensor with Kubios HRV Premium software, tracked closely to the reference device through an exercise ramp, illustrates the potential for this system to be of practical usage during endurance exercise.

## 1. Introduction

While respiratory frequency or breathing rate (RF) has been shown to be of value in monitoring both health and disease states, its application as an exercise measurement tool has lagged behind other internal and external load parameters such as heart rate (HR), heart rate variability (HRV), cycling, and running power [1]. However, there are scenarios in which RF estimation can be helpful in exercise science. These include ventilatory threshold measurements, assessments of work rate intensity, and decreases in exercise performance [2,3,4]. Though minute ventilation (the tidal volume x RF) has received attention for intensity estimation purposes [5], the respiratory rate itself has been shown to be quite sensitive in this regard as well [1]. Interestingly, the RF curve closely mimics that of the rise in lactate seen with progressive increases in exercise intensity [1]. It also appears to be a good predictor for constant intensity time to exhaustion and a means of differentiating effort/perceived exertion over long time periods [6]. Additionally, RF appears to respond to exercise load change faster than HR or VO_2_, making this metric ideal for high intensity interval physiologic tracking [7].

There are a wide variety of methods to determine RF, including formal gas exchange metabolic carts, devices that track mechanical deformation of the chest wall, sounds of breathing, and analysis of the photoplethysmogram (PPG) [8]. Unfortunately, both gas exchange carts and mechanical sensing vests can be cumbersome and costly. Photoplethysmography is problematic when used during dynamic exercise due to associated limb and body motion. However, it is possible to derive RF through the low-cost modality of HR monitoring technology [9]. One method is based on the alterations in HRV (as the variation of RR intervals) that accompany the process known as respiratory sinus arrythmia (RSA) [10]. On a simplistic basis, chest cavity expansion during inspiration induces an intrathoracic pressure drop with a secondary blood pressure reduction, leading to a reduced parasympathetic drive to the cardiac pacemaker apparatus causing heart rate to rise. Conversely, chest cavity volume contraction during expiration results in a return of parasympathetic drive and a slowing of HR [11]. Ideally, these cyclic changes in RR/HRV pattern are directly reflected to the RF and therefore measurable by HR monitoring or electrocardiogram (ECG) devices. In practice however, RF derived purely from RSA has relatively poor agreement with reference methods [9]. In an attempt to improve the accuracy of RSA-based RF, additional clues taken from the actual ECG waveform have been utilized to enhance those based on RSA alone. These methods involve several potential observations including the variation of R wave amplitude, QRS waveform analysis and/or QRS slope alteration during the breathing process [12,13,14].

Several reports have been published comparing the accuracy of different ECG and HRV algorithms to derive RF [13,15]. Many of these procedures are not easily reproduced by consumers, coaches, or sports professionals. Recently, one of the more popular commercial HRV software applications, Kubios HRV Premium, has been modified to calculate RF either using RR/HRV interval or ECG data recordings [16]. The HRV method is based on the cyclic cardiac beat to beat time domain changes in RR intervals associated with RSA, whereas the Kubios ECG procedure (ECG-derived RF; EDR) combines both the HRV estimation method with that of ECG-associated R wave amplitude changes seen during the respiratory cycle. To date, there has not been a published independent evaluation regarding the validity of these methods. Additionally, the question of whether adding the R wave amplitude information seen with ECG recording improves the RF estimation over simple RR interval analysis arises. In the context of comparing RF derived from two recording sources, it is also important to consider the effects of lead placement on HRV [17,18]. In other words, HRV measured from a conventional ECG lead placement may differ from that of HRV from a chest belt device. Fortunately, there are consumer HR monitoring devices with similar chest belt form factors able to accurately measure HRV and ECG waveforms, thus eliminating that particular variable. Therefore, to best compare HRV alone to that of HRV plus ECG-derived RF, we will compare data from two chest belt devices worn concurrently, the Polar H10 and Movesense Medical single-channel ECG, to a gas exchange-derived RF (GE).

## 2. Methods

### 2.1. Participants

Twenty-one participants (men: n = 12, age: 43 ± 13 years, height: 178 ± 8 cm, body weight: 83 ± 14 kg; women: n = 9, age: 35 ± 11 years, height: 169 ± 4 cm, body weight: 66 ± 10 kg) with no previous past medical history, current medications, or recent illness were recruited. They were all above 18 years of age and of any fitness level. All participants were asked to abstain from alcohol, caffeine, recreational drugs, tobacco, and vigorous exercise 24 h before testing, and provided written informed consent. Ethical approval for the study was acquired through the University of Hamburg, Department of Psychology and Movement Science, Germany (reference no.: 2021_400) and was in accordance with the principles of the Declaration of Helsinki.

### 2.2. Exercise Protocol and Data Recording

An incremental ramp protocol until exhaustion was performed on a mechanically braked cycle (Ergoselect 4 SN, Ergoline GmbG, Bitz, Germany) by all participants. Testing procedure included a warmup of three minutes with an initial workload of 50 watts then increasing by 1 watt every 3.6 s (equivalent to 50 watts/3 min). The exercise ramp was terminated when the participant could not maintain a cadence of 60 rpm or when they reached subjective exhaustion or a heart rate > 90% of the maximum predicted heart rate, or respiratory quotient > 1.1. Maximum oxygen uptake (VO_2MAX_) and maximum HR (HR_MAX_) were defined as the average VO_2_ and HR over the last 30 s of the test. Recordings of RR intervals and ECG were taken continuously with two devices at the same time, the Movesense Medical sensor (firmware version 2.0.99) single-channel ECG with chest belt (Movesense, Vantaa, Finland; sampling rate: 512 Hz; app software: Movesense Showcase version 1.0.9), and the Polar H10 sensor chest belt device (Polar Electro Oy, Kempele, Finland; sampling rate: 1000 Hz; app software: Elite HRV App, Version 5.5.1). Placement of both chest belt devices was just below the pectoral muscles with a similar horizontal alignment (see 18). Gas exchange kinetics including RF were recorded with a metabolic analyzer (Quark CPET, module A-67-100-02, Cosmed, Italy; desktop software: Omnia version 1.6.5). 

### 2.3. Data Processing 

RR data .txt files were exported from the Elite HRV app then processed by Kubios HRV Premium Software version 3.5 (Biosignal Analysis and Medical Imaging Group, Department of Physics, University of Kuopio, Kuopio, Finland). Movesense Medical sensor ECG tracings were recorded by the Movesense showcase app via an iPhone, converted into .csv files and also processed by Kubios HRV Premium. Preprocessing settings were set to the default values including the RR detrending method which was kept at “smoothness priors” (Lambda = 500). The RR series was then corrected by the Kubios HRV Premium “automatic method” [19]. For RF calculation, the window width was set to 30 s with a recalculation done every 1 s (grid interval = 1 s). Data sets with artefacts >3% were excluded from analysis. A 30 s window was based on recommendations from Kubios HRV [16]. A particular RF value was therefore based on the time 15 s before and 15 s after each given time stamp. The reference RF measured by the Quark CPET (breath by breath) was exported to Microsoft Excel 365 and time aligned with both the Polar H10 and Movesense Medical ECG sensor-derived RF. Since both the Polar H10 and Movesense Medical sensor ECG RF were recalculated every 1 s for both devices, only those values that time matched the gas exchange RF values were included for analysis.

### 2.4. Statistics

Normal distribution of data was checked by Shapiro–Wilk testing and visual inspection of data histograms. Descriptive statistical analysis was performed for the tested variables using Microsoft Excel 365 for the calculation of means, medians, and standard deviations (SD). The agreement of the derived RF during incremental exercise was assessed via linear regression, Pearson’s r correlation coefficient, coefficient of determination (R^2^), standard error of estimate (SEE), and Bland–Altman plots with limits of agreement (LoA) [20]. The size of Pearson’s r correlations was evaluated as follows: 0.3 ≤ r < 0.5 low, 0.6 ≤ r < 0.8 moderate, and r ≥ 0.8 high [21]. For non-normalized data, estimates of the adjusted median difference (AMD) were calculated using the Hodges–Lehmann shift method along with Wilcoxon testing of paired groups [22]. Agreement between groups was assessed by Bland–Altman analysis, but if proportional bias was detected, regression-based calculation of mean differences and limits of agreement were performed [23]. Bland–Altman mean differences for data comparisons were expressed as the absolute difference in RF as breaths/min (b/min). Inspection of the distribution of the mean differences in the Bland–Altman analysis was performed to confirm normality. For all tests, the statistical significance was accepted as *p* ≤ 0.05. Analytical statistics were performed using Microsoft Excel 365 with Real Statistics Resource Pack software (Release 7.6, copyright 2013–2021, Charles Zaiontz, www.real-statistics.com, accessed on 13 August 2022) and Analyse-it software (Leeds, UK, Version 6.01).

## 3. Results

During the incremental exercise ramp, participants achieved a mean VO_2MAX_ of 40.3 ± 7.9 mL/kg/min and HR_MAX_ of 176 ± 13 bpm, which was associated with a maximal power (P_MAX_) of 260 ± 53 watts. Five participants were excluded from exercise analysis due to artefacts > 3%. These were caused by both atrial and ventricular ectopic beats that were noted in the ECG. Artifacts attributed to noise were virtually nonexistent and ECG waveforms were well shaped in the analysis group. 

**Figure 1:**
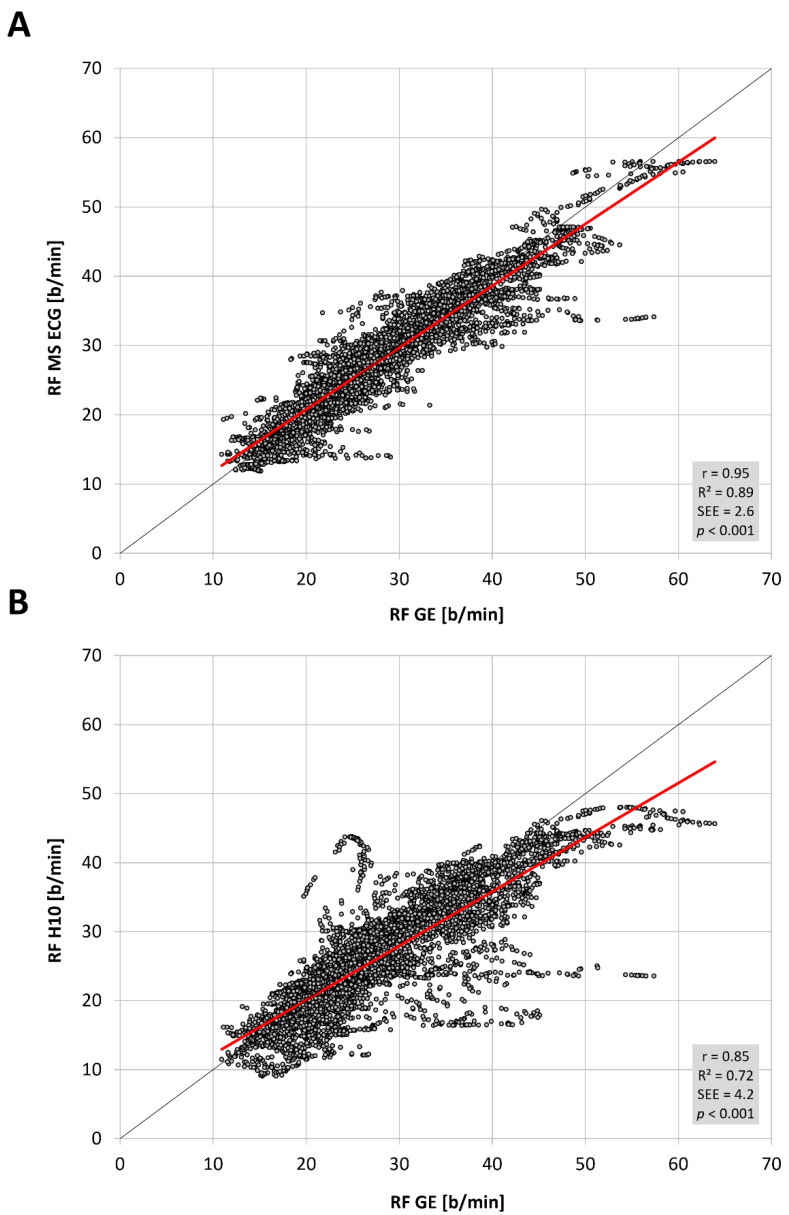
Regression plots for the comparison of respiratory frequency (RF) in breaths/min (b/min) for the (**A**) Movesense Medical sensor ECG (MS ECG) and the (**B**) Polar H10 sensor chest belt device (H10) vs gas exchange data (GE) during the incremental exercise test. Coefficient of determination (R^2^), Pearson’s r, standard error of estimate (SEE), and *p* value shown in the bottom right plot. Regression line in red, line of unity shown in dark grey.

**Figure 2:**
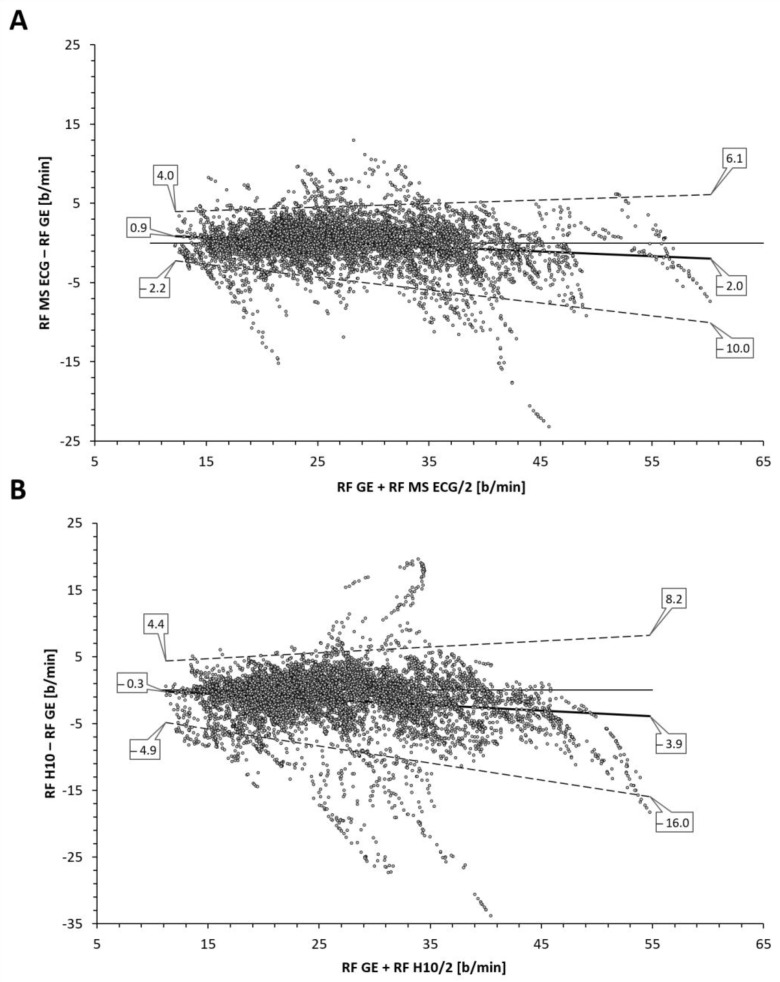
Bland–Altman analysis of respiratory frequency (RF) as breaths/min (b/min) for the (**A**) Movesense Medical sensor ECG (MS ECG) and the (**B**) Polar H10 sensor chest belt device (H10) vs the gas exchange data (GE) during the incremental exercise test until voluntary exhaustion. Center solid line in each plot represents the mean bias (difference) between each paired value as absolute values. The top and bottom dashed lines are LoA (1.96 standard deviations from the mean difference).

**Figure 3:**
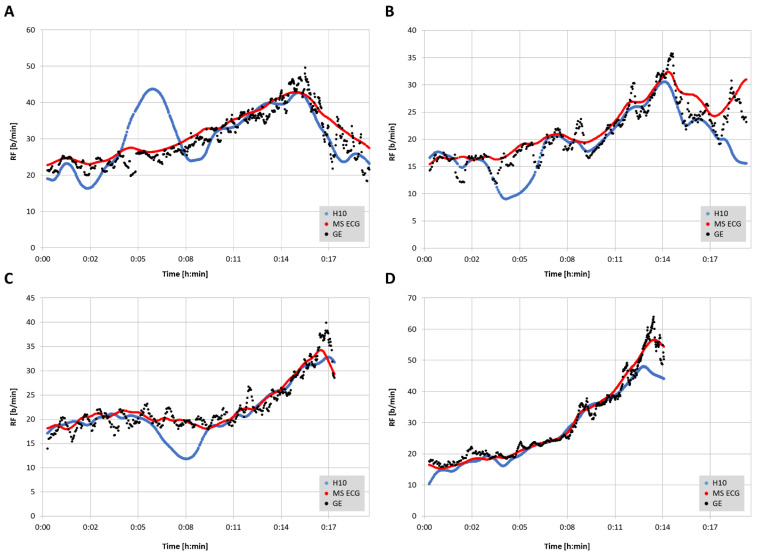
Respiratory frequency (RF) plotted over time for Movesense Medical sensor ECG (MS ECG), Polar H10 (H10) (Kubios window width: 30 s, grid interval: 1 s) and the gas exchange data (GE) in four representative participants. (**A**): 26-year-old female with a BMI of 30 kg/m², VO_2MAX_ of 38 mL/kg/min, Kubios artifact rate of 0.5%; (**B**): 27-year-old female with a BMI of 22 kg/m², VO_2MAX_ of 42 mL/kg/min, Kubios artifact rate of 0.0%; (**C**): 47-year-old male with a BMI of 38 kg/m², VO_2MAX_ of 31 mL/kg/min, Kubios artifact rate of 0.0%; (**D**): 25-year-old female with a BMI of 21 kg/m², VO_2MAX_ of 40 mL/kg/min, Kubios HRV artifact rate of 0.7%. MS ECG (red circle), H10 (blue circle), GE (black). Ramp termination corresponds with peak GE respiratory rate. Length of post ramp recovery determined by data recording cessation and artifacts below 3%.

The total number of paired RF observations between devices was 7543 from 16 participants and the distribution of values was not normal. The level of correlation was high (Figure 1, Table 1) between the reference gas exchange device and both the Polar H10 (r = 0.85, SEE = 4.2) and Movesense Medical ECG sensor (r = 0.95, SEE = 2.6). Although median values were statistically different via Wilcoxon testing, adjusted median differences were clinically small for the Polar H10 (RF about 1 b/min) and trivial for the Movesense Medical ECG sensor (RF about 0.1 b/min). Bland–Altman plotting is shown in Figure 2. An analysis looking for both proportional bias (change in the bias over the RF range) and heteroscedasticity (change in scatter of the differences) did show significant findings for each comparison. A line of regression for the mean differences and limits of agreement was performed and displayed in Figure 2 according to the recommendations of Ludbrook [23]. Representative plots of RF over time for the three measurement modalities are shown in Figure 3. In 2 of the cases there were zero artifacts and in the other 2 the total artifacts (atrial premature beats) were below 1%. 

## 4. Discussion

The aim of this study was to assess the level of agreement for RF detection between a reference gas exchange analyzer and either RR interval/HRV analysis alone (Polar H10) or a combination of RR interval analysis and ECG waveform fluctuation (Movesense Medical ECG sensor) using a commercial software application, Kubios HRV Premium. The findings show that RF derived from a combination of both RR intervals and ECG were closer to the reference values than from RR intervals only. Both correlation coefficients, adjusted median differences, Bland–Altman bias and LoAs were superior for the combination approach. In both methodologies, diminished accuracy appears to occur at higher respiratory rates and/or at the ramp termination with voluntary exhaustion. In almost all cases, this under reporting of RF was more prominent with the RR interval only method. However, for the most part, both absolute values as well as the shape of the RF over time curve were well preserved with the combination RR intervals and ECG data.

Inspection of the Bland–Altman plots did reveal significant proportional bias and heteroscedasticity (change in scatter of the differences) with both methods. With the RR interval-based approach the mean bias varied from −0.3 to −3.9 b/min along with relatively wide LoA (maximum 8.2 to −16.0 b/min). Although the AMD was only about 1 b/min, there was a general failure to properly measure the higher RF with precision as well as more frequent outliers than with the combination approach. Improvement in agreement and correlation was seen using the combination of RR intervals and ECG data with a reduction of mean bias variation (0.9 to −2.0 b/min), LoA range (maximum 6.1 to −10.0 b/min) and a trivial AMD. Both r, R2, and SEE were superior with this method but even this algorithm still failed to fully capture the highest RF portions at ramp termination at voluntary exhaustion. This disparity in values at high RF was also seen with Kubios’ own internal white paper report [16]. Their data did mirror the present findings in showing that the combination approach was superior to the RR interval only method especially in the high RF zone. 

It should be noted that the Movesense Medical ECG sensor and the Polar H10 should display virtually identical base ECG waveforms, since they use the same subpectoral sensor pad placement. As background information, the Polar H10 is capable of transmitting ECG waveform data at a fixed sample rate of 130 Hz with several android and iOS applications available to read this data. This distinction is important as other studies regarding HRV indexes have shown differences based purely on the ECG lead chosen for comparison [17,18]. In the present case, each device had similar sensor electrode placements, suggesting that this issue should not be a concern. Both devices also had high sample rates at 512 and 1000 Hz, respectively, both above recommended levels [24]. In another report [14], a Polar H10 ECG waveform was upsampled (from 130 to 1000 Hz) and analyzed for RF using a combination of RR interval and ECG morphology change during various participant activities including running and cycling. Although correlations and participant specific plotting were not shown, the Bland–Altman differences were minimal with a bias of −0.5 and LoA of 2 b/min. The authors did report that the error rate was higher during running than with cycling.

From a practical standpoint, the EDR seems to yield equivalent RF patterns as the GE noted in Figure 3. Since previous studies showed potential for RF breakpoints to correspond with ventilatory thresholds [2], it would be of interest to see if EDR could achieve a similar result. A recent publication showed that both first and second ventilatory threshold identification is possible with EDR methodology [25]. This study used a Holter monitor ECG with a sample rate of 1000 Hz, and lead V6. Although we did not attempt to correlate gas exchange thresholds with the shape of the RF curve, prototypical RF over time plots shown in the cited report display many similarities to that of the Movesense Medical ECG sensor seen in Figure 3. Unfortunately, in many cases from the current report that used RR interval information only, the RF over time plot had skewed regions that would make breakpoint estimation difficult. Another endurance exercise characteristic that could be examined with EDR is the ability of RF to act as an index of “acute performance decrement (APD)” [4] as a consequence of training load. The APD appears to be a similar concept to that of athletic “durability” described as the time of onset and magnitude in deterioration in physiological-profiling characteristics over an exercise session [26]. Given the high concordance between EDR and GDR seen in this report, it seems plausible that EDR could be substituted for more equipment intensive measurements of RF. In the study overview by Passfield et al. [4], the APD was well correlated with the RF, supporting the potential of this metric to follow exercise load effects.

## 5. Limitations and Future Directions

Several potential limitations are apparent in using both RR interval related RSA patterns and/or ECG morphology for the purpose of RF estimation. The precision of RR measurement, amount of noise or artifact will certainly play a role in accurate delineation of any measure of HRV [27,28,29] and presumably derived RF as well. However, in addition to these concerns, the ECG based algorithm can be hampered by poor waveform signal strength, highlighting the need for optimal sensor pad/chest belt placement. In a similar fashion, even RR measurement can be affected by lead placement and waveform morphology, as noted. To compound matters, the induction of body motion and muscular contractile effects will make a suboptimal waveform even more difficult to parse. It is interesting to note that in the subjects with deviation in the RR interval-derived RF seen in Figure 3, the addition of the ECG algorithm caused almost complete correction of the abnormal tracking. It is also important to realize that the data presented here represent a best-case scenario, with excellent ECG waveforms, little-to-no noise/missed beat artifacts and rare premature beats. The effects of both cardiac arrythmia, missed beats, and noisy ECG tracings are unclear. We strongly suggest that individuals inspect their ECG waveforms before testing to optimize QRS morphology (to achieve best R peak voltage) and signal-to-noise ratio. It is also of note that few validations have been performed investigating EDR during high intensity activity, let alone incremental exercise ramps until voluntary exhaustion [14]. In the future, a promising area of technology involving wearable, washable fabric sensors may provide a solution to ECG-related noise and arrhythmia issues in respiratory monitoring as well [30]. Additionally, progress in compensating for motion artifacts in PPG-derived indexes [31] may lead to better accuracy in forthcoming applications related to RF calculation [32].

The present study was performed with participants cycling indoors. Extrapolation to either outdoor cycling or other sport modalities (running, row, ski) needs to be made with caution until further validation is done. Some evidence points to subtle changes in the patterns of RF between exercise modalities such as cycling, running, and rowing that are dependent on entrainment effects [32,33,34,35]. In addition to issues related to exercise modality, higher EDR error rates were observed in participants running rather than cycling [14]. Since that specific study used a similar chest belt sensor (Polar H10), similar preprocessing and EDR methodology to Kubios HRV Premium software (Pan Tompkins R peak identification with quantification of R peak voltage combined with RR interval timing), this may indicate some limitation of using the current implementation with certain sporting activities. Regarding RF data point matching in the current study, the RR interval/EDR values were calculated over a measurement window of 30 s. It is possible that the failure to fully reach the peak RF seen with the gas exchange reference device may be related to the limited time duration of that RF. We also did not time-average the gas exchange values, which, if executed, may have led to less scattering of differences on the Bland–Altman plots. Some recommendations have been made to time-average the RF to remove effects of swallows, coughs, and sighs [1]. It was felt, that for the most part, breathing patterns during a cycling ramp would contain little of the above. Our intent was to directly compare devices with as little data manipulation as possible. Finally, the issue of device specific HRV precision should not be a factor since both the Polar H10 [36] and the Movesense Medical ECG sensor [18] have had formal RR validation studies performed.

Despite the above considerations, the degree of similarity between the EDR and GDR were impressive. Except for the slight underrepresentation of maximal values at ramp termination at voluntary exhaustion, the overall shape and agreement of the incremental rise of RF values were clinically meaningful. In the context of insights into exercise intensity assessment and threshold demarcation, the RF values seen with the Movesense Medical sensor ECG were virtually indistinguishable to that of the reference device values. With a similar form factor to a conventional chest belt monitor and only a minimal additional cost, the Movesense Medical sensor ECG appears very promising for athletic RF estimation in conjunction with Kubios HRV Premium software. One additional consideration is the cost of Kubios HRV Premium which is required for both ECG interpretation and any RF analysis. Beyond price, what are the prospects for future wearable devices (watches, cycling head units) to include incorporation of ECG-derived RF into dedicated apps which record from the Movesense Medical ECG sensor directly? Although this may appear to be unrealistic given the hardware and software constraints of mobile units, the accomplishment of real time computation of the nonlinear HRV index DFA a1 for the purpose of athletic monitoring by several apps [37] illustrates what is potentially possible with skillful software design.

## 6. Conclusions

The ability of a commercial HRV software package, Kubios HRV Premium, to estimate respiratory frequency throughout an exercise ramp was assessed in two consumer heart rate monitoring devices, the Polar H10 and Movesense Medical ECG sensor. Bland–Altman analysis, linear regression, and adjusted median differences indicate that the ECG centric system (single-channel chest belt ECG plus Kubios HRV Premium ECG algorithm) is superior to that of RR interval-derived respiratory frequency. The ECG based methodology also captured the pattern and shape of the respiratory frequency rise over time during the incremental ramp, whereas the RR interval-based system displayed variable accuracy especially at high exercise intensities. Future confirmation of these findings needs to be carried out with other exercise modalities as well as evaluation of the effects of artifact and noise. However, the use of commercially available software and hardware for the purpose of respiratory frequency monitoring appears promising.

## Figures and Tables

**Table 1:** Mean, standard deviation (SD), median, minimum, maximum, adjusted median difference (AMD) as breaths/min (b/min) for the respiratory frequency (RF) comparison of the gas exchange (GE), Polar H10 (H10) and the Movesense Medical sensor ECG (MS ECG) data according to Hodges–Lehmann method (p-value estimated by Wilcoxon paired testing), Pearson’s r and standard error of estimate (SEE) calculated from paired RF data during the incremental exercise test until voluntary exhaustion.

	GE	H10	MS ECG
Mean (b/min)	27.75	26.19	27.70
Median (b/min)	25.80	25.09	26.51
SD (b/min)	8.56	7.92	8.08
Max (b/min)	63.93	48.01	56.57
Min (b/min)	10.91	9.06	11.89
AMD (b/min)		−1.159	0.105
Wilcoxon *p* value		0.0001	0.004
Pearson’s r		0.85	0.95
SEE (b/min)		4.2	2.6

## Data Availability

The raw data supporting the conclusions of this article will be made available by the authors, without undue reservation.

## Abbreviations

ECG:	Electrocardiogram
EDR:	ECG derived respiration
HRV:	Heart rate variability
GE:	Gas exchange
PPG:	Photoplethysmogram
RF:	Respiratory frequency
RR interval:	Time between 2 successive R peaks in the ECG
RSA:	Respiratory sinus arrythmia
